# *Daxx* Variation as a Potential Predictive Marker of the Therapeutic Response to Neoadjuvant Chemoradiotherapy in Locally Advanced Rectal Cancer

**DOI:** 10.1002/cam4.70815

**Published:** 2025-03-25

**Authors:** Xi Zhu, Xiaoming Kao, Leilei Liu, Xuan Wang, Yang Li, Qiurong Li

**Affiliations:** ^1^ Research Institute of General Surgery, Jinling Hospital, Affiliated Hospital of Medical School Nanjing University Nanjing China; ^2^ Research Institute of General Surgery, Jinling Hospital Nanjing Medical University Nanjing China; ^3^ Department of Pathology, Jinling Hospital, Affiliated Hospital of Medical School Nanjing University Nanjing China

**Keywords:** *Daxx*, locally advanced rectal cancer, neoadjuvant chemoradiotherapy, predictive biomarker, therapeutic response

## Abstract

**Objective:**

The response to neoadjuvant chemoradiotherapy (NACRT) for locally advanced rectal cancer (LARC) varies from achieving a complete pathological response to encountering resistance to treatment. Therefore, biomarkers for predicting the NACRT responses should be identified. This prospective study aimed to identify key genomic biomarkers as the predictors of the NACRT response with LARC.

**Methods:**

Overall, 67 patients with LARC treated with NACRT and proctectomy were divided into two groups based on the tumor regression grade (TRG) for identifying key biomarkers. Patients with a TRG of 0 or 1 were assigned to the sensitive response group, and patients with a TRG of 2 or 3 were the resistant response group. Twenty‐nine postsurgical tumor samples were collected for whole exome sequencing (WES) to identify genomic variation biomarkers. The other 38 pairs of tumor specimens from pretreatment and postsurgery samples were evaluated by immunohistochemistry (IHC) to examine the biomarker features.

**Results:**

In the WES subcohort, 11 genes showed copy number variation, including *FNKBIA*, *ARID1A*, *CCND2*, *CDK4*, *LYN*, *MDM2*, *RAD51B*, *RARA*, *SPEN*, *STAT3*, and *Daxx*, which has the highest copy number variation. For the IHC subcohort, *Daxx* was initially highly expressed in the nuclei of tumor cells, particularly in the sensitive response group, while varying its expression after NACRT, demonstrating that *Daxx* levels were related to treatment responses and the survival benefit, especially a better disease‐free survival (DFS).

**Conclusion:**

We identified multiple genomic variations between sensitive and resistant responders and verified that *Daxx* is a potential predictive biomarker of the response to NACRT in LARC.

## Graphical abstract

Multiple genomic variations between sensitive and resistant responders to NACRT in LARC were found. High *Daxx* expression is a potential predictive biomarker for the response to NACRT and is associated with a favorable clinical prognosis.

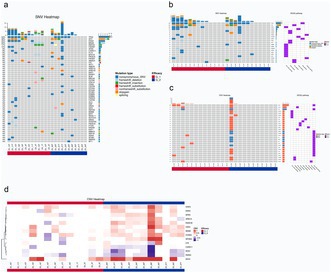

## 1 Introduction

At present, colorectal cancer (CRC) ranks fourth and second in the global incidence of malignant tumors and cancer‐related mortality [1]. As shown in the epidemiology by 2022, about 4,824,700 new cancer cases occurred in China [2]. Due to the development of comprehensive treatment strategies, the survival of CRC patients has improved, and the standard regimens for locally advanced rectal cancer (LARC) are neoadjuvant chemoradiotherapy (NACRT) followed by low anterior resection and total mesorectal excision (TME) to reduce local recurrence rates and improve survival [1, 3]. With the continuous progress of oncology comprehensive research, the new adjuvant treatments of LARC have improved: The mode of radiotherapy has developed from long‐course radiotherapy (LCCRT) to short‐course radiotherapy (SRT) by penetrating tumor tissues and inducing cytotoxic damage to proliferating cells through direct and indirect mechanisms [4], or the transformation of chemotherapy from induction chemotherapy to enhancing radiotherapy sensitization to systemic consolidation chemotherapy such as total neoadjuvant therapy (TNT). Even technological innovations from external radiotherapy to intracavitary irradiation like the OPERA study have occurred to improve the treatment efficiency for patients [5, 6, 7]. However, even under similar histopathological features, different therapeutic efficacies follow NACRT owing to tumor heterogeneity. Approximately 20%–40% of patients show a pathological complete response (pCR), correlated with better disease‐free survival (DFS) and overall survival (OS) rates than those who do not reach a pCR [8, 9]. Still, up to 40% of patients experience a minimal to poor treatment response after NACRT [10]. Therefore, to raise efficiency with NACRT and improve survival, some studies have explored new schemes such as neoadjuvant‐intensified radiochemotherapy [11] or combining with immunological treatments. In 2004, Habr‐Gama et al. [12] first reported the results of the watch‐and‐wait approach following a complete clinical response (cCR) to NACRT, and patients could choose organ‐preservation therapy under strict surveillance after cCR. Therefore, imaging characteristics such as DW‐MRI or clinical features such as serum biomarkers are predictive factors for preoperative NACRT responses but still not enough [13]. Thus, predictable markers are needed to effectively select patients expected to exhibit poor responses to NACRT before treatment to formulate personal regimens.

Several microarray analyses demonstrated that the expression of some genes affected radiosensitivity and played high predictive accuracies [14, 15, 16]. However, these studies yielded few overlapping findings, resulting in less effective radiosensitizers for enhancing neoadjuvant treatment responses. Then, whole exome sequencing (WES) significantly advanced the field of cancer treatments and partly elucidated the complex landscapes of malignancies. The variants or errors of the exome were found to acquire clonal diseases, such as tumors or Mendelian diseases [17]. Next‐generation sequencing techniques also helped advance research on rectal cancer [18, 19], and several somatic mutations are identified as therapeutic targets or prognostic markers, including *AKT1*, *APC*, *BRAF*, *KRAS*, *NRAS*, *PIK3CA*, *PTEN*, *SMAD4*, *TGFBR2*, *TP53*, and microsatellite instability [20, 21, 22]. However, these biomarkers do not effectively predict NACRT response in LARC.

In this study, we evaluated genetic variation in patients with LARC undergoing NACRT to identify specific genomic biomarkers related to the treatment response and radiosensitivity. Immunohistochemistry (IHC) was performed to detect the expressions of notable biomarkers in order to estimate the general features and potential predictive or prognostic value.

## 2 Materials and Methods

### 2.1 Study Design and Patients

In total, 67 patients with LARC who underwent NACRT and proctectomy at the Jinling Hospital of Nanjing Medical University (Nanjing, China) between June 2015 and December 2020 were recruited prospectively. Based on the differences in the obtained tumor specimens, two subcohorts were further determined (prior WES and IHC subcohorts). In the prior WES subcohort, surgical tumor specimens and matched blood samples were collected from 29 patients after neoadjuvant therapy, and genomic DNA was extracted from tumors and peripheral blood lymphocytes for WES sequencing. In the IHC subcohort, pretreatment tumor endoscopic biopsies and post‐treatment surgical samples were collected from 38 patients for IHC analyses.

The inclusion criteria were as follows: (1) all patients had endoscopy biopsy‐proven adenocarcinoma within 12 cm of the anal verge; (2) tumors were preoperatively staged as LARC (stage cT3/T4 or cT any cN1/2, cM0) using pelvic magnetic resonance imaging as well as thoracic and abdominal‐pelvic enhanced computed tomography; and (3) TME was performed after NACRT completion with LCCRT. The exclusion criteria were as follows: (1) clinical tumor‐node‐metastasis (TNM) stages I and IV; (2) synchronous diagnosis of other carcinomas; (3) obstruction, perforation, bleeding, and palliative surgery; and (4) patients underwent the watch‐and‐wait strategy or refused to accept treatment. Pathological specimens were reviewed in serial sections, and the TRG was evaluated using the American Joint Committee on Cancer (AJCC) criteria by two independent pathologists. All patients received preoperative LCCRT, with 45–50.4 Gy in 25–28 fractions to the pelvis, combined with oral capecitabine (825 mg/m^2^, twice daily, 5 days/week). The standard TME surgeries were performed 6–12 weeks after completion of NACRT, followed by adjuvant CAPOX with 6 cycles.

The patients were divided into a sensitive response group (G_1) and a resistant response group (G_2) based on the TRG. Patients with a TRG of 0 or 1 were assigned to the sensitive response group (G_1), while patients with a TRG of 2 or 3 were assigned to the resistant response group (G_2). Patients were followed up until death or the cutoff date (July 31, 2022) every 6 months for the first 3 years after surgery and yearly thereafter. The study was approved by the Ethics Committee of Jinling Hospital (Approval no. 2022DZKY‐051‐01), and all patients signed written consent forms.

### 2.2 DNA Mutation Analysis

After extracting genomic DNA from tumors and peripheral blood lymphocytes, the DNA concentration was measured using Qubit dsDNA HS (High Sensitivity) Assay Kit (Themo Fisher, Lenexa, KS, USA), and DNA quality was assessed using an Agilent 2100 BioAnalyzer (Agilent, Santa Clara, CA, USA). The library was constructed using the KAPA Hyper Preparation Kit (Kapa Biosystems, Wilmington, MA, USA) and quantified using the AccuGreen High Sensitivity dsDNA Quantitation Kit (Biotium, Fremont, CA, USA). The final sequencing libraries were quantified using a Qubit dsDNA HS Assay Kit (Thermo Fisher). Paired‐end sequencing on captured samples was performed on an Illumina NovaSeq6000 (Illumina, San Diego, CA, USA). After data quality control via Trimmomatic (V0.36), reference mapping using BWA aligner (V0.7.17), and duplication masking using Picard (V2.23.0, GitHub Inc., USA). After data quality control via Trimmomatic (V0.36) to remove adaptor sequences and low‐quality bases, clean reads were mapped to the human reference genome (hg19) using BWA (V0.7.17), followed by sorting and duplication masking with Picard (V2.23.0). The Genome Analysis ToolKit (V3.7) was used for realignment. Finally, the processed BAM file was obtained and used for subsequent analysis.

### 2.3 Variant Calling

Single‐nucleotide variants (SNVs) were identified using VarDict (V1.5.1), whereas compound heterozygous mutations were merged using FreeBayes (V1.2.0). After ANNOVAR annotations, somatic mutations are selected if the following standards are not met: (1) located in intergenic regions or intronic regions, (2) synonymous SNVs, (3) allele frequency ≥ 0.002 in the Exome Aggregation Consortium (ExAC) and genomAD databases, (4) allele frequency < 0.05 in the tumor sample and allele frequency < 0.01 in the plasma sample, (5) strand bias mutations in the reads, (6) support reads < 5, and (7) depth < 30. Copy number variations (CNVs) were compared using CNVkit (V0.9.2) between tumor and normal samples, with a copy number threshold of 3 to identify CNV gains, and a threshold of 1.2 was used to detect CNV losses.

### 2.4 Immunohistochemistry

Four‐micrometer‐thick tumor sections were stained with *Daxx* rabbit polyclonal antibody at a 1:100 dilution (#YT1293; ImmunoWay Biotech, Plano, TX, USA) on 38 pairs of formalin‐fixed paraffin‐embedded (FFPE) specimens in the IHC subcohort. The IHC analysis was initially performed by two experienced pathologists using the following nuclear staining IHC scores: 0 = no staining, 1 = 1%–5%, 2 = 6%–25%, 3 = 26%–75%, 4 = 76%–100% of tumor cells. A MoticEasyScan system (Motic China Group Co. Ltd., Xiamen, China) and Motic Digital Slide Assistant Lite 1.0 (Motic China Group Co., Ltd.) were used for scanning the images of total slide regions. Image Pro Plus 6 (IPP, Media Cybernetcs Co., USA) was used to semi‐quantitatively analyze the IHC image data for *Daxx* expression with the mean optical density (MOD).

### 2.5 Statistical Analyses

A Kaplan–Meier analysis was used to estimate DFS and OS. Comparisons were made using the log‐rank test. Univariate and multivariate survival analyses were performed using logistic regression and Cox proportional hazard models. Fisher's exact test and Wilcoxon test were used to analyze differences in the log_2_ ratio of copy numbers for gene mutations. A paired *t*‐test was used to compare the MOD of IHC data. SPSS V.26.0 (IBM Corp, Armonk, NY, USA), GraphPad Prism V.9.0 (GraphPad Software, La Jolla, CA, USA), and the R system were used for data analyses and the generation of graphs. Statistical significance was set at *p* < 0.05.

## 3 Results

The clinicopathological features of the WES and IHC subcohorts are listed in Table 1. Of the 67 surgical specimens, 14 (21%) demonstrated a pCR. Of these, five were in the WES subcohort and nine were in the IHC subcohort, respectively.

**TABLE 1:** Clinicopathological features of prospective cohorts.

Features	Prospective cohorts (*n* = 67)					
	WES subcohort			IHC subcohort		
	(*n* = 29)			(*n* = 38)		
	Sensitive G_1 group	Resistant G_2 group	*p*	Sensitive G_1 group	Resistant G_2 group	*p*
	(*n* = 18)	(*n* = 11)		(*n* = 15)	(*n* = 23)	
**Age (median, IQR)**	69, 55–67	63, 56–64	0.881	54, 53–69	73, 55–66	0.986
**Gender, *n* (%)**			1.000			1.000
Male	11 (60.1)	6 (54.5)		11 (73.3)	17 (73.9)	
Female	7 (38.9)	5 (45.5)		4 (26.7)	6 (26.1)	
**Pretreatment cT stage, *n* (%)**			1.000			0.832
cT1‐cT2	3 (16.7)	2 (18.2)		6 (40.0)	10 (43.5)	
cT3‐cT4	15 (83.3)	9 (81.8)		9 (60.0)	13 (56.5)	
**Pretreatment cN stage, *n* (%)**			0.277			0.501
cN0	4 (22.2)	0 (0)		4 (26.7)	9 (39.1)	
cN1‐2	14 (77.8)	11 (100)		11 (73.3)	14 (60.9)	
**Pretreatment tumor size, *n* (%)**			1.000			0.832
> 4 cm	8 (44.4)	5 (45.45)		6 (40.0)	10 (43.48)	
≤ 4 cm	10 (55.6)	6 (54.55)		9 (60.0)	13 (56.52)	
**Differentiation status, *n* (%)**			0.694			1.000
Well and moderate	13 (72.2)	7 (63.6)		13 (86.7)	19 (82.6)	
Poor and undifferentiated	5 (27.8)	4 (36.4)		2 (13.3)	4 (17.4)	
**Pretreatment CEA level (ng/ml), median, (range)**	2.34 (1.00–24.96)	2.536 (0.84–12.58)	0.582	4.16 (1.03–12.53)	3.70 (1.16–25.45)	0.877
**ypT stage, *n* (%)**			0.044			< 0.001
pCR	5 (27.8)	0 (0.0)		9 (60.0)	0 (0.0)	
pTis and pT1‐2	9 (50.0)	4 (36.4)		1 (6.7)	12 (52.2)	
pT3‐4	4 (22.2)	7 (63.6)		5 (33.3)	11 (47.8)	0.440
**ypN stage, *n* (%)**			0.237			
pN0	14 (77.8)	6 (54.5)		13 (86.7)	17 (73.9)	
pN1‐2	4 (22.2)	5 (45.5)		2 (13.3)	6 (26.1)	
**TRG, *n* (%)**			< 0.001			< 0.001
0 (pCR)	5 (27.8)	0 (0.0)		9 (60.0)	0 (0.0)	
1	13 (72.2)	0 (0.0)		6 (40.0)	0 (0.0)	
2	0 (0)	6 (54.5)		0 (0.0)	11 (47.8)	
3	0 (0)	5 (45.5)		0 (0.0)	12 (52.2)	

### 3.1 Aberrations and Predictive Variables of the WES Subcohort

**FIGURE 1:**
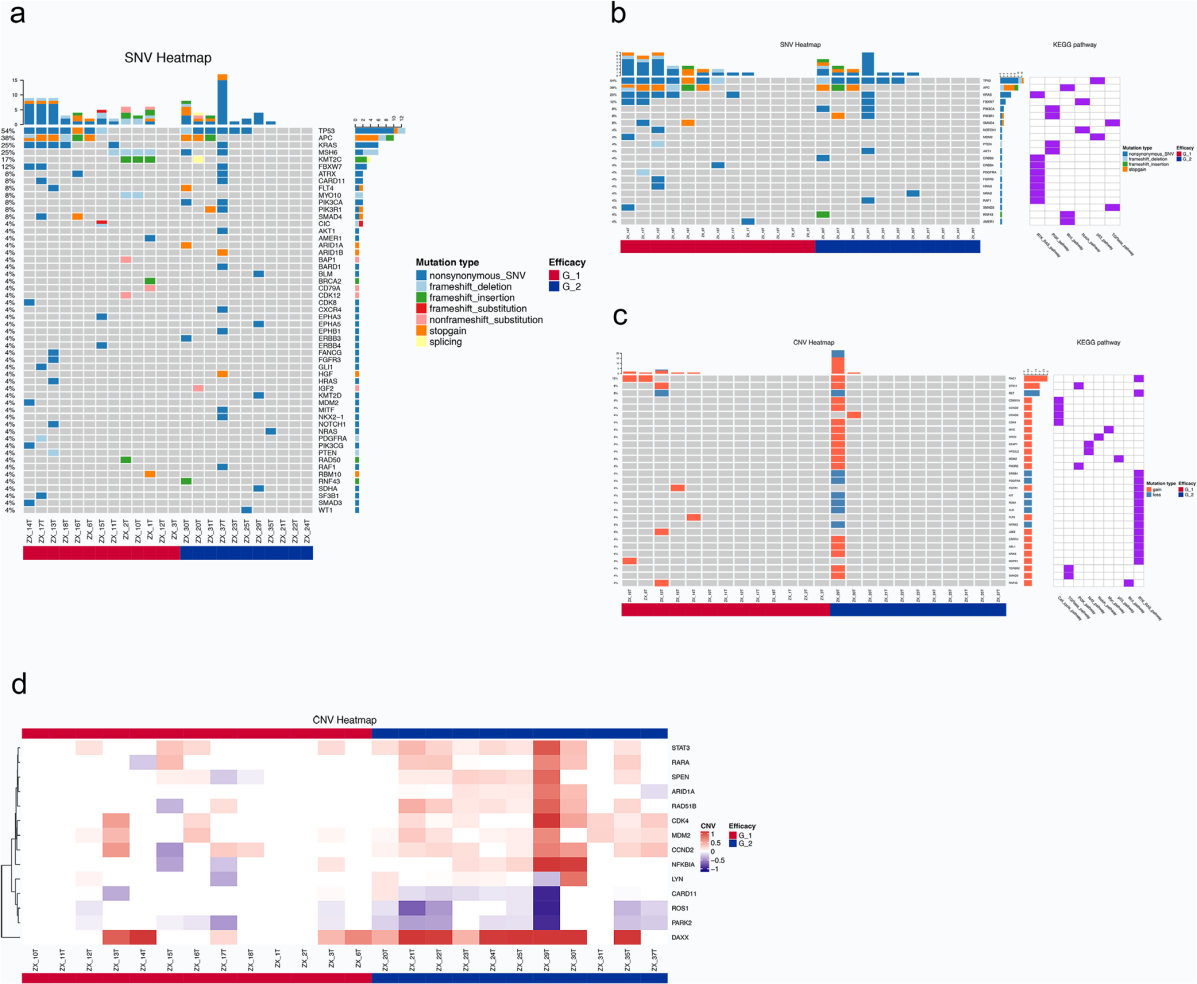
Genetic mutations in the WES subcohort after NACRT differing between the sensitive response group (G_1) and the resistant group (G_2). (a) Overall mutational landscape of SNVs for patients; (b) Pathway enrichment analysis of genes with SNVs; (c) Pathway enrichment analysis of genes with CNV; (d) Landscape of differently expressed CNVs between the two groups.

DNA mutation analysis was performed to investigate the distribution of variations in 29 surgical samples from the WES subcohort, and global gene features were identified using NGS. As shown in Figure 1, after eliminating five pCR samples with no remaining tumor tissue, 13 and 11 tumor samples remained in the sensitive response (G_1) and resistant response groups (G_2). The SNV map shows somatic mutations with a population frequency of > 4%. Tumor protein 53 (*TP53*) had the highest mutant frequency at 54%, followed by adenomatous polyposis coli (*APC*) (38%), Kirsten rat sarcoma viral oncogene homolog (*KRAS*) (25%), mutS homolog 6 (*MSH6*) (25%), Lysine Methyltransferase 2C (*KMT2C*) (17%), and F‐box and WD repeat domain containing 7 (*FBXW7*) (12%).

Only six signaling pathways were detected via KEGG pathway enrichment analysis, and mutations were mainly in the RTK‐RAS pathway. However, no significant difference was found between the two groups. CNV analysis revealed 96 genes with copy number aberrations, and KEGG pathway analysis revealed nine major tumor‐related signaling pathways with CNVs, mainly in the RTK‐RAS pathway. Among these, 14 genes showed significant differences, as determined by the Wilcoxon test between the two groups, with 11 genes showing copy number gains, including Death Domain‐Associated Protein (*Daxx*), NF‐Kappa‐B Inhibitor Alpha (*FNKBIA*), AT‐Rich Interaction Domain 1A (*ARID1A*), Cyclin D2 (*CCND2*), cyclin‐dependent kinase 4 (*CDK4*), LYN proto‐oncogene (*LYN*), Mouse double minute 2 homolog (*MDM2*), RAD51 Paralog B (*RAD51B*), retinoic acid receptor, alpha (*RARA*), spen family transcriptional repressor (*SPEN*), and Signal transducer and activator of transcription 3 (*STAT3*). Additionally, there were three gene losses, including Caspase Recruitment Domain Family Member 11 (*CARD11*), parkin RBR E3 Ubiquitin Protein Ligase (*PARK2*), and ROS proto‐oncogene 1 (*ROS1*). Notably, *Daxx* had the highest number of CNVs in G_2 (64%) and G_1 (23%). *Daxx* displays an important role in biological processes and oncogenesis; a high CNV of *Daxx* in G_2 may be related to the poor NACRT response or treatment resistance. To this end, we hypothesized that genomic variation in *Daxx* might be correlated with TRG and even the LARC survival with NACRT.

### 3.2 Relationship Between *Daxx* Expression and TRG in the IHC Subcohort

**FIGURE 2:**
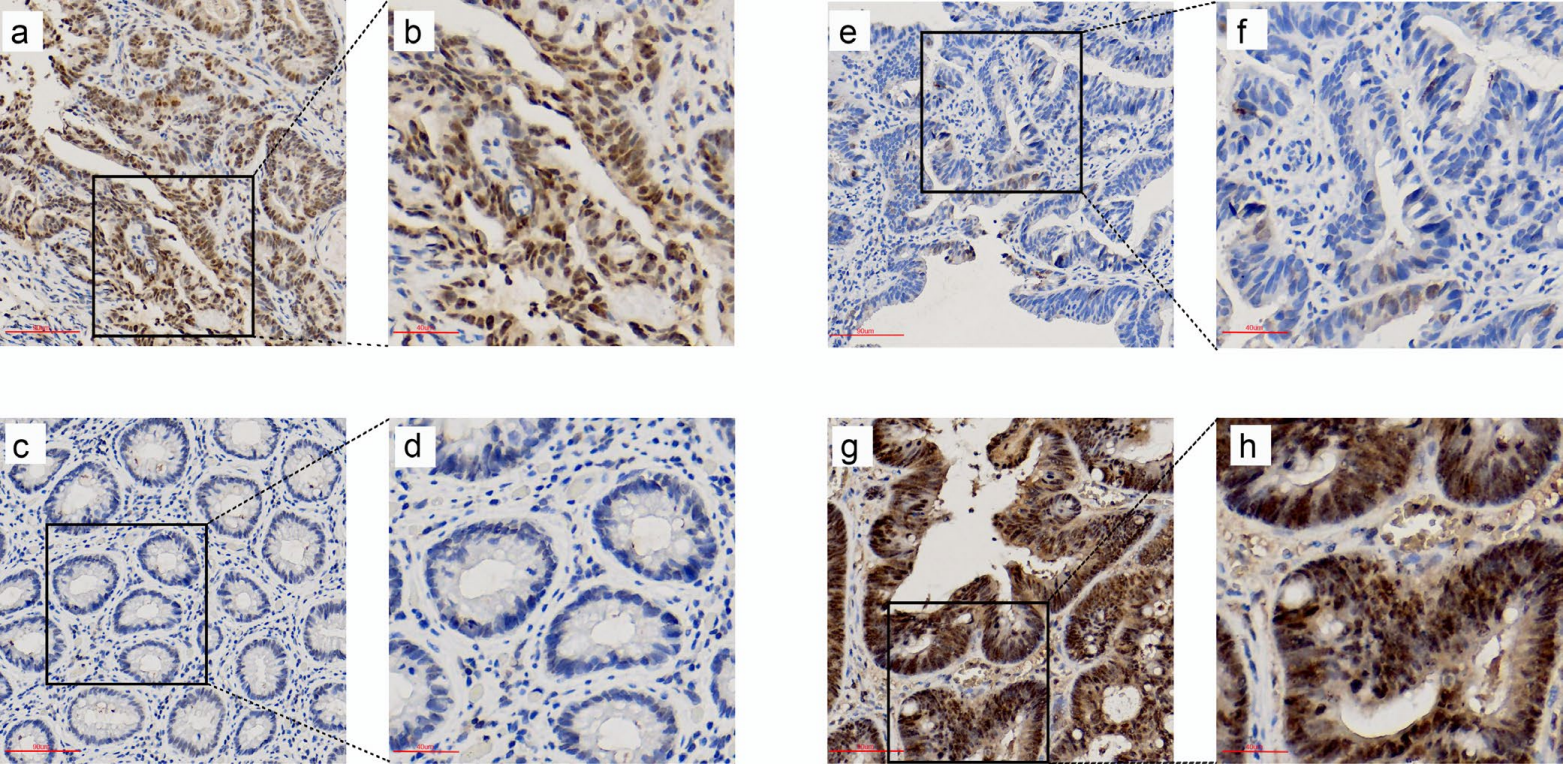
Expression of Daxx in tumor tissues before and after NACRT. (a) Daxx was highly expressed in the nucleus in the sensitive response group (G_1) before treatment at 20× magnification; (c) Daxx was not expressed in G_1 with TRG 0 after NACRT at 20× magnification; (e) Little Daxx was expressed in the nucleus pretreatment in the resistant response group (G_2) at 20× magnification; (g) Daxx was highly expressed both in the nucleus and in the cytoplasm post‐treatment in G_2 at 20× magnification; (b, d, f, h) Visual field measured at 400× magnification.

Thirty‐eight pairs of matched FFPE specimens, including pretreatment tumor endoscopy biopsies and post‐treatment surgical samples, were analyzed using IHC. As shown in Figure 2, before NACRT, *Daxx* was expressed in 37/38 (97%) specimens, and staining features were predominantly detected in the nucleus Notably, the nucleus staining scores were as follows: 0, 1, 2, 3, and 4 for 1, 5, 6, 15, and 11 specimens pretreatment, which showed the *Daxx* nuclear staining score before NACRT was associated with TRG (*p* = 0.0035).

**FIGURE 3:**
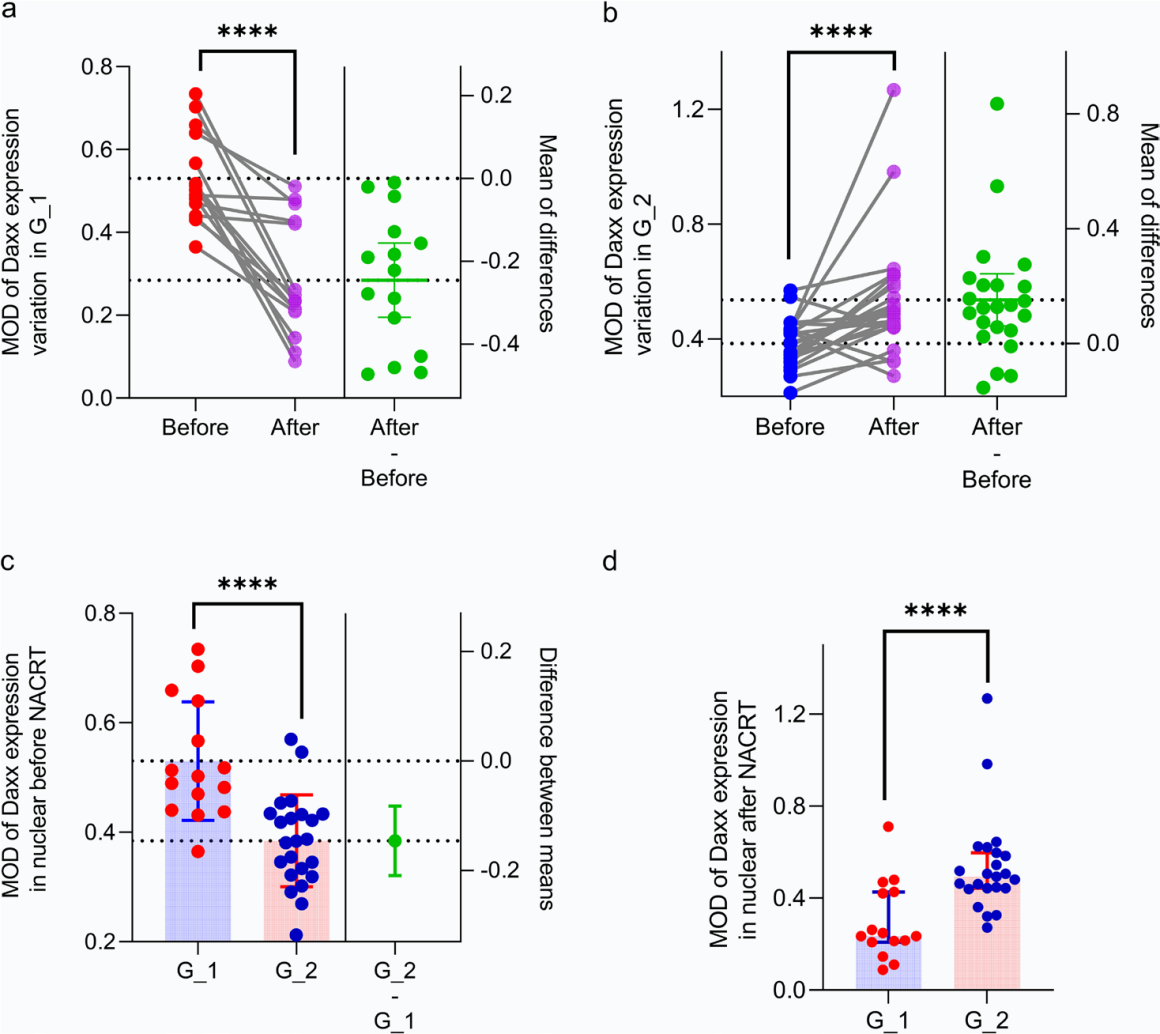
Positive nuclear MOD values of Daxx expression. (a) MOD values of Daxx expression in G_1 were high pretreatment and decreased post‐NACRT; (b) MOD of Daxx expression in G_2 was extremely low pretreatment and increased post‐NACRT; (c) MOD values of Daxx expression pretreatment. The expression levels in G_1 were considerably higher than in G_2; (d) MOD values of Daxx expression after NACRT. The expression levels in G_1 were considerably lower than those in G_2. ****Mean *p* < 0.0001.

After analyzing the MOD data in Figure 3, there were significant differences in *Daxx* expression between the two groups before and after NACRT. *Daxx* in pretreatment tumor tissues had higher MOD values in G_1 than MOD values in G_2, and MOD values decreased after NACRT owing to more tumor regressions in TRG 0 and TRG1. In contrast, the tumor tissues in G_2 had low *Daxx* MOD values before treatment, but the post‐treatment MOD values increased with less tumor shrinkage. Also noteworthy, in TRG 3, the initial MOD values for *Daxx* expression in G_2 were very low or nonexistent, indicating poor TRG or treatment response. Overall, the pretreatment expression of *Daxx* in G_1 was much higher than in G_2, whereas the *Daxx* expression in G_2 was increased after NACRT.

**FIGURE 4:**
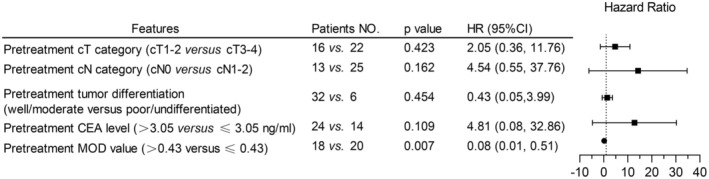
Multivariate logistic regression of clinicopathological parameters for tumor regression in the IHC subcohort. The pretreatment MOD values of Daxx were related to TRG.

Following a receiver operating curve analysis, a Youden index value of 0.43 was selected as the pretreatment cutoff value to categorize *Daxx* high and low expression. As shown in Figure 4, the high expression of *Daxx* was an independent predictor of TRG (HR 0.08; 95% CI = 0.01–0.51, *p* = 0.007). As described above, *Daxx* was located in the nucleus and was more highly expressed in G_1 than in G_2 before NACRT. After treatment, the expression of *Daxx* increased in G_2, but decreased with the tumor regression in G_1, indicating the variation in *Daxx* in the IHC subcohort was consistent with the CNV analysis in the WES subcohort.

### 3.3 Survival Analysis and *Daxx* Expression

**FIGURE 5:**
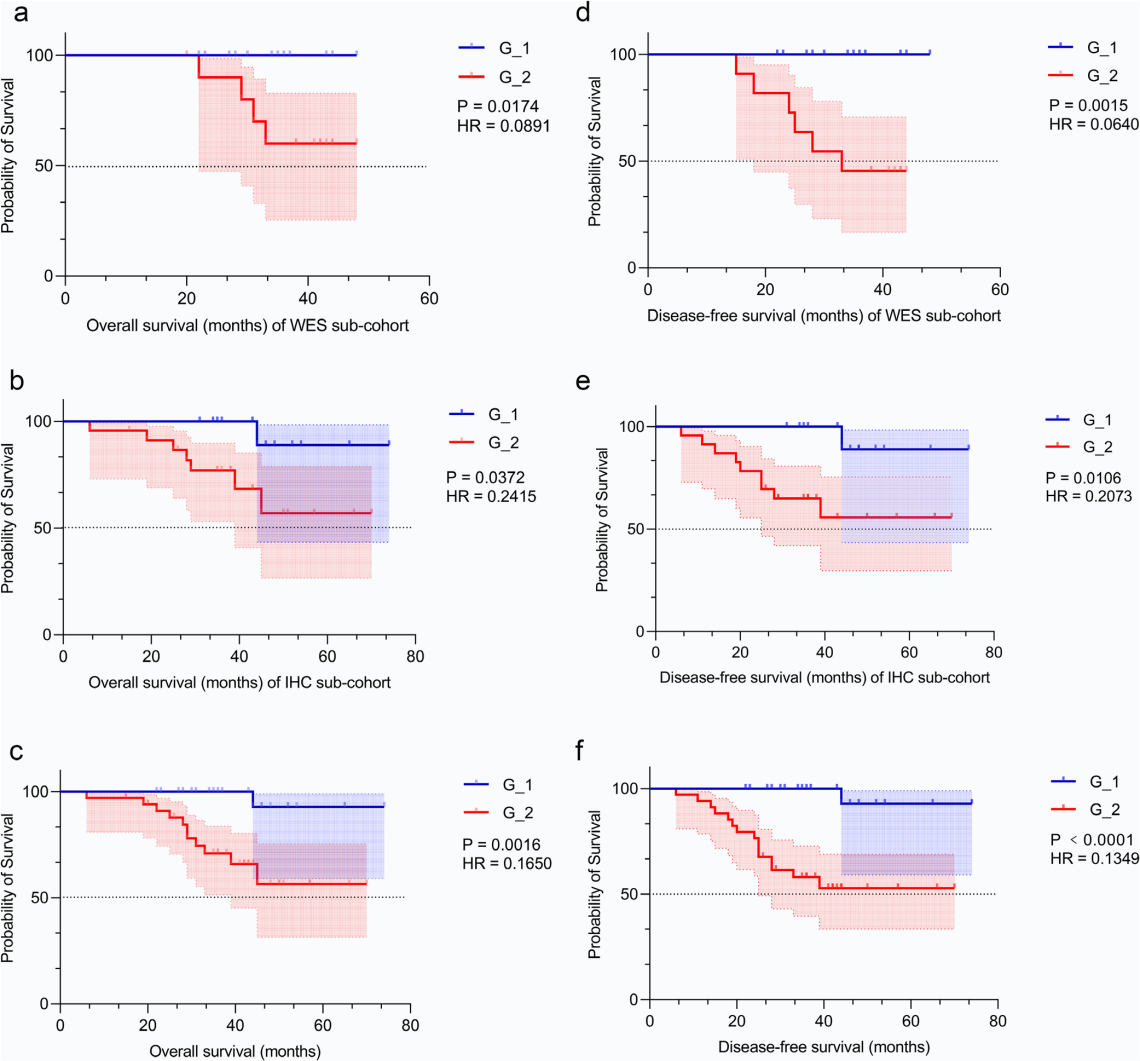
OS and DFS of the WES subcohort, IHC subcohort, and all patients. (a) OS for WES subcohort; (b) OS for IHC subcohort; (c) OS for all patients in two cohorts; (d) DFS for WES subcohort; (e) DFS for IHC subcohort; (f) DFS for all patients.

**FIGURE 6:**
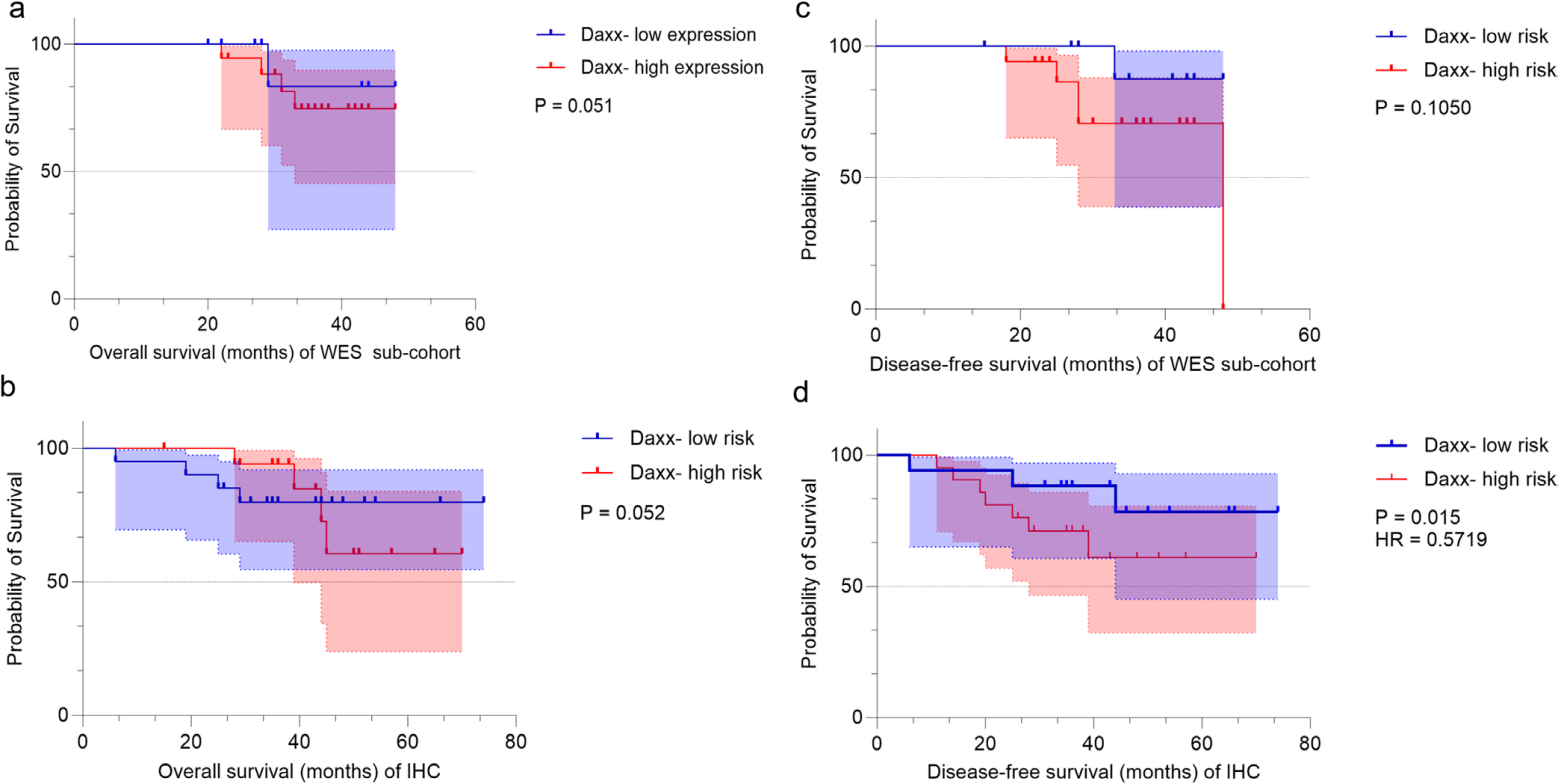
OS and DFS of the WES subcohort, IHC subcohort with high‐risk patients. (a) OS for WES subcohort with Daxx CNVs expression; (b) OS for IHC subcohort with high‐risk, the cutoff Daxx mod value = 0.43; (c) DFS for WES subcohort with Daxx CNVs expression; (d) DFS for IHC subcohort with high‐risk, the cutoff Daxx mod value = 0.43.

Figure 5 shows the survival analysis of all patients. After a median follow‐up of 38 months (range: 6.1–75 months), the Kaplan–Meier curve revealed that G_2 had a shorter DFS (HR = 0.1349, 95% CI = 0.04987–0.3650, *p* < 0.0001) and OS (HR = 0.1650, 95% CI = 0.05286–0.5153, *p* = 0.0016) than G_1 in all cohorts, suggesting a significantly different prognosis between the two groups. In G_1, 2/33 (6.1%) of patients relapsed locally, compared with 15/34 (44%) of patients who relapsed locally, developed distant metastases, or died in G_2. Moreover, 2/33 (6.1%) of patients died in G_1, compared with 11/34 (32.4%) of patients who died related to rectal cancer in G_2. The probability of 3‐year DFS and OS for all patients was 76.4% and 83.2%, respectively. However, we used the *Daxx* CNVs and Youden index value with 0.43 of MOD value to define high‐risk patients successively to explore the relationship with survival and found high expression with *Daxx* was associated with DFS (Figure 6). A Cox regression analysis revealed that the TRG and neoadjuvant pathological stage (ypTNM) were associated with DFS and OS, and pretreatment MOD value was associated with DFS (Table 2).

**TABLE 2:** Cox regression analysis of Daxx expression and clinicopathological covariates with survival in the IHC sub‐cohort.

	Disease‐free survival				Overall survival			
	Univariate analysis		Multivariate analysis		Univariate analysis		Multivariate analysis	
	HR (95% CI)	*p*	HR (95% CI)	*p*	HR (95% CI)	*p*	HR (95% CI)	*p*
Age (> 54 versus ≤ 54 years)	0.67 (0.14, 3.18)	0.615	0.34 (0.032 3.66)	0.373	0.71 (0.14, 3.60)	0.678	0.41 (0.03, 6.20)	0.523
Gender (M versus F)	2.321 (0.49, 10.9)	0.287	1.55 (0.07, 33.38)	0.778	1.58 (0.32, 7.83)	0.576	6.49 (0.11, 398.70)	0.373
Pretreatment tumor size (> 4 versus ≤ 4 cm)	0.75 (0.22, 2.58)	0.643	0.723 (0.11, 4.84)	0.740	0.69 (0.17, 2.78)	0.606	0.41 (0.03, 6.20)	0.523
Pretreatment cT category (cT1‐2 versus cT3‐4)	1.25 (0.36, 4.33)	0.723	0.51 (0.03, 9.17)	0.650	1.24 (0.31, 4.96)	0.761	0.39 (0.02, 8.93)	0.553
Pretreatment cN category (cN0 versus cN1‐2)	1.72 (0.48, 6.11)	0.402	0.73 (0.03, 18.45)	0.851	1.64 (0.39, 6.88)	0.503	0.42 (0.01, 17.09)	0.645
Pretreatment tumor differentiation (well/moderate versus poor/undifferentiated)	0.42 (0.11, 1.65)	0.216	4.74 (0.13, 171.51)	0.395	0.26 (0.06, 1.12)	0.071	0.27 (0.01, 9.92)	0.476
Pretreatment CEA level (> 3.05 versus ≤ 3.05 ng/mL)	1.74 (0.51, 6.01)	0.383	3.05 (0.52, 18.07)	0.219	1.50 (0.37, 6.65)	0.566	1.94 (0.24, 15.92)	0.536
Pretreatment MOD value (> 0.43 versus ≤ 0.43)	0.00 (0.00, 1.16)	0.046	0.03 (0.00, 173.83)	0.043	0.02 (0.00, 9.43)	0.213	4.56 (0.00, 212.828)	0.782
TRG	2.99 (1.30, 6.89)	0.010	7.44 (1.11, 50.08)	0.039	2.39 (1.04, 5.483)	0.039	12.90 (1.34, 124.44)	0.027
Post‐treatment ypT category (T1‐2 versus T3‐4)	1.96 (1.03, 3.73)	0.041	1.42 (0.21, 9.66)	0.721	1.56 (0.78, 3.120)	0.210	0.15 (0.02, 1.488)	0.106
Post‐treatment ypN category (N0 versus N1‐2)	3.28 (1.45, 7.45)	0.005	8.70 (1.30, 58.40)	0.026	5.00 (1.96, 12.720)	0.001	9.00 (1.40, 57.98)	0.021
Post‐treatment MOD value (> 0.43 versus ≤ 0.43)	8.21 (0.75, 89.61)	0.041	1.05 (0.01, 233.25)	0.986	1.87 (0.16, 21.59)	0.618	8.208 (0.752, 89.61)	0.084

## 4 Discussion

This study was designed to identify genomic biomarkers that could effectively predict the NACRT response and survival in patients with LARC. Genomic signatures are associated with tumorigenesis, disease progression, treatment resistance, and prognosis [20, 21, 22]. According to the pathogenesis, the occurrence and development of CRC can be roughly divided into three types: chromosomal instability (CIN), microsatellite instability (MSI), and CpG Island Methylation Phenotype (CIMP) [23, 24]. CIN is mainly manifested as structural abnormalities of chromosomes and aneuploidy, chromosome deletion, and rearrangement, which lead to chromosomal variation, various gene mutations, and promote the occurrence of tumors [25]. Studies suggest some somatic mutations act as potential markers affecting NACRT treatment sensitivity and survival prognosis of LARC patients. High *EREG* expression can predict a better CRT response, which is associated with an early pre‐Tx and post‐Tx tumor status in rectal cancer [26]. *VSTM2L* overexpression in CRC causes CRT resistance by affecting cell proliferation and apoptosis [27]. As major prognostic cell types in rectal cancer, inflammatory cancer‐associated fibroblasts improve therapeutic response and long‐term survival [28].

Our studies found that, in the WES subcohort, *Daxx* showed the most significant difference in copy number variation frequency between the treatment‐sensitive group and the resistance group, with the highest variation frequency in postoperative tissue specimens of the resistance group, suggesting that copy number amplification of *Daxx* may be negatively correlated with NACRT efficacy and TRG grading. Considering *Daxx* genomic variants may be associated with tumor regression or with changes in the initial status of tumor cells via NACRT in the WES subcohort, additional IHC paired tests were performed to determine the features of *Daxx* before and after NACRT. Further studies showed that *Daxx* was commonly expressed in the nucleus in LARC tissues, and the expression of *Daxx* is related to the therapeutic effect of NACRT. Higher expression of *Daxx* predicts better TRG grading, leading to a better NACRT treatment response, suggesting that higher expression of *Daxx* benefits the TRG grade and survival.

*Daxx*, as a Fas receptor death domain‐associated protein, mediates the activation of apoptosis signal‐regulating kinase 1 through the Jun N‐terminal kinase pathway [29, 30], and recent studies show it also plays an important regulatory role in the development of oncology [30]. Structurally, *Daxx* consists of two helical bundles (4HB) containing a defined binding surface for ATRX, RASSF1C, p53, and MDM2. It also includes a histone‐binding domain in complex with an H3.3/H4 dimer; two SIM motifs and an acid domain appear to increase the H3.3/H4 dimer binding affinity [30]. In previous studies, *Daxx* is highly expressed in a variety of human malignant tumors confirmed by IHC, including prostate cancer [31], ovarian cancer [32], oral squamous cell carcinoma [33], and gastric cancer [34]. Liu et al. [35] found that in patients with liver metastasis, *Daxx* was highly expressed in colorectal tumor tissues, and the expression of *Daxx* in liver tissues was lower than that in primary colon cancer tissues. Experiments showed that *Daxx* shows the cancer inhibitory effect by interacting with ZEB1 and directly inhibiting the activity of the E‐cadherin promoter in a ZEB1‐dependent manner. Via the CD24 or β‐catenin pathway, *Daxx* plays a tumor suppressor role by regulating the biological processes in human tumor samples and in vitro [36]. Another clinical study suggested that the expression of *Daxx* in colorectal adenocarcinoma cells was lower than that in normal colon tissue cells [37]. Huang et al. [38] reported that the expression of *Daxx* was increased in both clinical CRC samples and colorectal cell lines. In the xenotransplantation model, *Daxx* knockout significantly reduced the proliferative activity of CRC cells and tumor growth, suggesting that *Daxx* played a role in promoting cancer in CRC.

Besides, whether *Daxx* plays a tumor‐suppressive or oncogenic role in rectal cancer and the potential mechanisms for radiosensitivity remain unclear. Jason et al. [39] recently analyzed the genomic sequence of tumor samples to compare genomic variations in LARC with CRT in patients who had achieved a pCR to chemoradiation versus a poor response, and they found *Daxx‐ZBTB22* mutations in poor responders but no mutation occurred in complete responders, indicating *Daxx* participates in oncogenesis and radiation resistance. In ovarian cancer, increased *Daxx* expression promotes DNA repair and protects cancer cells from DNA damage‐induced cell death, leading to resistance to both chemotherapy and X‐ray radiation lesions [30]. After *Daxx* downregulation in primary fibroblasts, cells resist cell death induced by both UV irradiation and oxidative stress. Activation of the JNK pathway is significantly impaired in *Daxx*‐depleted cells upon both UV and H_2_O_2_ exposure, similar to the upstream JNK kinase MKK4/SEK1, indicating that *Daxx* has regulatory functions upstream of JNK [29]. Taken together, *Daxx* may act as a radiosensitizer to enhance tumor regression.

Particularly, *Daxx* was expressed less or rarely in the nucleus of rectal tumor cells before NACRT in the resistant group, but was increased post‐treatment, assuming *Daxx* relocalization or reproduction was possibly associated with chemoradiotherapy. *Daxx* interacts with various proteins with functions in the nucleus and cytoplasm, and is mainly localized in the nuclei of tumor cells to mediate antiapoptosis [30]. Furthermore, in gastric cancer, *Daxx* is mainly expressed in the nucleus, while it can be found both in the cytoplasm and in the nucleus in intestinal metaplasia of the gastric mucosa, indicating that the nuclear/cytoplasmic *Daxx* ratio may be a prognostic factor for the response to chemotherapy and survival [34]. Our results revealed a remarkable difference in *Daxx* expression and its changes with NACRT between the sensitive and resistant LARC response groups, indicating *Daxx* may act as a potential predictor of the NACRT response and reflect a potential radiosensitizer.

In this study, all patients completed the LCCRT, and better responses or TRG were closely correlated with prolonged survival. The 3‐year DFS probability was similar to that observed in the Chinese FOWARC study [6], and *Daxx* was associated with DFS of LARC. Recent studies have found that SRT for LARC has higher treatment compliance and tolerability than that of LCCRT, while it has lower probabilities of disease‐related treatment failure and lower acute toxicities, resulting in higher rates of pCR and longer survival [40, 41]. Thus, we hypothesize that patients with low *Daxx* expression might benefit from SRT to achieve better outcomes.

Our study has limitations. First, the sample size of the prospective cohort was small, and a larger number of samples should be recruited to characterize the genomic patterns and evaluate the predictive power of biomarkers. Second, further studies are warranted to confirm the hypothesis and identify potential mechanisms.

## 5 Conclusions

In summary, multiple genomic variations were observed between NACRT‐sensitive and resistant groups in patients with LARC. *Daxx* was more highly expressed in sensitive patients than in the resistant patients prior to NACRT. After NACRT, *Daxx* expression increased in the resistant group compared with levels before treatment, and this phenomenon potentially reflects a novel mechanism of radiosensitivity. All patients with sensitive responses showed better survival, and a pretreatment higher expression led to a longer DFS, indicating that *Daxx* may be a potential predictive biomarker for NACRT response associated with a better clinical prognosis.

## Author Contributions

**Xi Zhu:** conceptualization (equal), data curation (lead), formal analysis (equal), investigation (lead), writing – original draft (equal). **Xiaoming Kao:** conceptualization (equal), resources (equal), writing – original draft (equal). **Leilei Liu:** formal analysis (equal), methodology (equal), writing – review and editing (equal). **Xuan Wang:** formal analysis (equal). **Yang Li:** investigation (equal), methodology (lead), writing – original draft (equal), writing – review and editing (lead). **Qiurong Li:** conceptualization (equal), methodology (supporting), project administration (supporting), resources (equal), writing – review and editing (lead).

## Ethics Statement

All procedures performed in studies involving human participants were in accordance with the ethical standards of the Jinling Hospital of Nanjing Medical University (2022DZKY‐051‐01) and with the 1964 Helsinki declaration and its later amendments or comparable ethical standards.

## Conflicts of Interest

The authors declare no conflicts of interest.

## Data Availability

The datasets used in this study are available from the corresponding author upon reasonable request.
